# Synergistic efficacy of phage Henu10 with antibiotics against *Shigella dysenteriae* with insight into phage resistance and fitness trade-offs

**DOI:** 10.3389/fcimb.2024.1499325

**Published:** 2024-12-06

**Authors:** Jing Zhao, Baohong Chen, Weizhen Wang, Yu Kang, Erli Hu, Yuan Zhang, Huiling Chen, Xiao Xu, Xinying Ji, Yuhan Wang, Tieshan Teng, Salwa E. Gomaa

**Affiliations:** ^1^ Gynaecology Department, Hua County People’s Hospital, Anyang, China; ^2^ Institute of Biomedical Informatics, School of Basic Medical Sciences, Henan University, Kaifeng, China; ^3^ Department of Microbiology and Immunology, Faculty of Pharmacy, Zagazig University, Zagazig, Egypt

**Keywords:** *Shigella dysenteriae*, shigellosis, antibiotic resistance, phage therapy, phage-resistance, phage-antibiotics synergy, fitness cost

## Abstract

**Introduction:**

The irrational use of antibiotics has facilitated the emergence of multidrug- resistant *Shigella spp*., undermining the effectiveness of the currently available antibiotics. Consequently, there is an urgent need to explore new approaches, with phage therapy emerging as a promising alternative.

**Methods:**

In this study, we isolated a phage targeting Shigella dysenteriae from sewage samples using DLA methold, designated Henu10. The morphology, biological characteristics, genomic composition, and phylogenetic relationships of Henu10 were thoroughly characterized. To investigate the trade-off relationship between phage resistance and bacterial fitness, phage Henu10-resistant strains R6 and R11 were identified using continuous passage and bidirectional validation methods.

**Results:**

Phage-resistant strains R6 and R11 exhibited impaired adsorption, increased sensitivity to temperature and pH stress, heightened susceptibility to certain antibiotics (such as ciprofloxacin and kanamycin), reduced biofilm-forming capacity, and diminished colonization ability in vivo compared to the wild-type strain.

**Discussion:**

These results indicate that phage Henu10 may effectively control the pathogenic bacteria associated with *S. dysenteriae*, representing a promising new therapeutic option for treating *S. dysenteriae* infections.

## 1 Introduction

*Shigella* species, a gram-negative bacillus, is responsible for the acute diarrheal infection known as bacillary dysentery or shigellosis, which is a significant public health concern in many developing countries (Shears, 1996). *Shigella* is commonly transmitted through fecal-oral routes or person-to-person contact, with increased susceptibility among the very young, elderly, and immunocompromised. The *Shigella* infectious dose is remarkably low, with as few as 10 organisms potentially causing illness. *Shigella* is resistant to gastric acid and able to survive passage through the stomach to enter the intestine. Approximately 140 million people are affected by shigellosis, with an estimated 600,000 deaths occurring annually on a global scale (Vaccine research and development, 1997; Niyogi, 2005; Hausdorff et al., 2023). Shigellosis is associated with four serogroups of *Shigella*, namely serogroup A (*Shigella dysenteriae*), serogroup B (*Shigella flexneri*), serogroup C (*Shigella boydii*), and serogroup D (*Shigella sonnie*) (Peleg et al., 2014; Jamal et al., 2015). Infections resulting from *S. dysenteriae* type 1 typically lead to the most severe form of dysentery, often with life-threatening complications (Sreenivasan et al., 2013).

Antimicrobial resistance is a pressing global public health issue that severely affects the effectiveness of modern medical care and antimicrobial interventions against pathogenic bacteria, including *Shigella*. Several antibiotics have been used to treat shigellosis since the beginning of the antibiotic era, including ampicillin, nalidixic acid, tetracycline, chloramphenicol, and trimethoprim-sulfamethoxazole. However, the development of resistance to these antibiotics led to the use of ciprofloxacin, azithromycin, and ceftriaxone. Despite this, the effectiveness of these treatments has also been compromised by the emergence of resistant *Shigella* spp (Klontz and Singh, 2015). The WHO has listed *Shigella* as one of the priority pathogens for which new antibiotics are urgently needed (Shrivastava et al., 2018). New antibiotic development is challenging, and there is no effective vaccine currently available to prevent shigellosis. Consequently, alternate approaches are required, such as phage therapy (Deris et al., 2013).

Phage therapy has recently been considered a potential solution for addressing antibiotic-resistant bacterial infections. Phages were first used by d’Herelle in 1919 to treat four instances of dysentery at the Children’s Hospital in Paris (Chanishvili, 2012). Phages offer numerous substantial advantages over conventional antibiotics. Nevertheless, bacteria can also develop resistance to phages during therapy. Consequently, the combined use of phages and antibiotics could help mitigate resistance development. Phage-antibiotic synergy (PAS) phenomenon describes the use of phages with sub-lethal concentrations of antibiotics that can work better together than each agent alone, leading to potential synergy (Li et al., 2021a). There are several mechanisms underlying PAS, including the cell filamentation effect induced by β-lactam antibiotics; reduced occurrence of resistance to either phages or antibiotics alone; enhanced antibiotic susceptibility; the reduction of antibiotic MIC; increased plaque size, phage amplification, and burst size; and the phage-produced enzymes, which facilitate antibiotic diffusion and cell penetration (Łusiak-Szelachowska et al., 2022).

PAS has been recently reported to enable an evolutionary trade-off (Gordillo Altamirano et al., 2021). Recent studies investigating the fitness trade-offs between phages and antibiotic resistance have unveiled novel prospects for the advancement in phage therapy (Oechslin, 2018; Mangalea and Duerkop, 2020; Oromí-Bosch et al., 2023). The emergence of phage-resistant bacteria could help restore susceptibility to antibiotics and decrease bacterial virulence (Fujiki et al., 2023), thereby enhancing the scientific understanding of phage therapy in conjunction with antibiotics in clinical practice that maximizes the beneficial effects of the trade-off relationship (Mi et al., 2023). The current study involved the isolation and characterization of a newly isolated lytic phage, Henu10, as well as developing an approach for treating shigellosis utilizing a combination of phage and antibiotics. Additionally, screening and isolation of phage-resistant strains, evaluating their response to various stressors, conducting virulence assessment, and evaluating the *in vivo* fitness using a mouse model compared to the wild-type strain.

## 2 Results

### 2.1 Phage morphology and the TEM analysis

**Figure 1:**
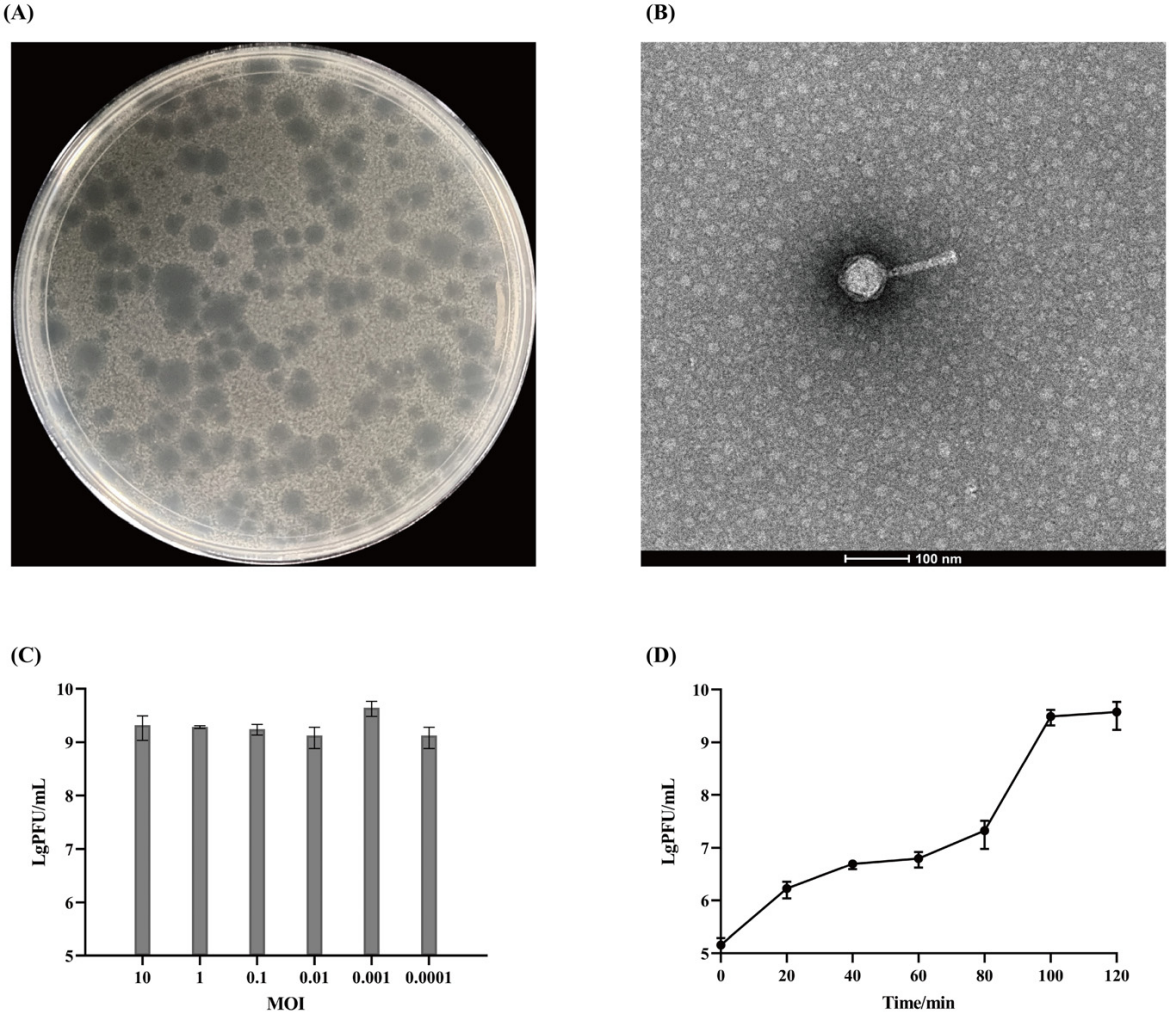
The morphology and biological characteristics of phage Henu10. **(A)** The plaque morphology of phage Henu10 on a double layer agar plate. **(B)** The electron microscopy of phage Henu10 particles. **(C)** Optimal multiplicity of infection (MOI) of phage Henu10. **(D)** One-step growth experiment. PFU, plaque forming unit.

On nutrient agar plates, phage Henu10 exhibits circular, clear plaques with diameters of approximately 2 mm. The plaques were surrounded by translucent halo zones (**Figure 1A**). Phage particles morphology were observed using TEM revealing that isolated phage has an icosahedral head of about 50 ± 2 nm in diameter and a contractile tail of 100 nm in length (**Figure 1B**).

### 2.2 Biological characteristics of phage Henu10

The highest phage titer, 3.3×10^9^ PFU/mL, was observed when the MOI was set at 1:1000, indicating this is the OMOI of phage Henu10 (**Figure 1C**). The one-step growth curve was conducted, which revealed that during the initial 0-80 min, there was minimal change in phage titer, indicating the latent period of phage Henu10. Subsequently, between 80-100 min, there was an exponential increase in phage titer, signifying the burst period. After reaching its peak around 100-120 min, no further changes were observed during the plateau period. By applying the outbreak quantity formula for phage Henu10: outbreak quantity = final cleavage product concentration/initial host bacterial concentration, the average burst size is 30 PFU per infected cell (**Figure 1D**).

### 2.3 Stability of phage Henu10 against diverse environmental conditions

**Figure 2:**
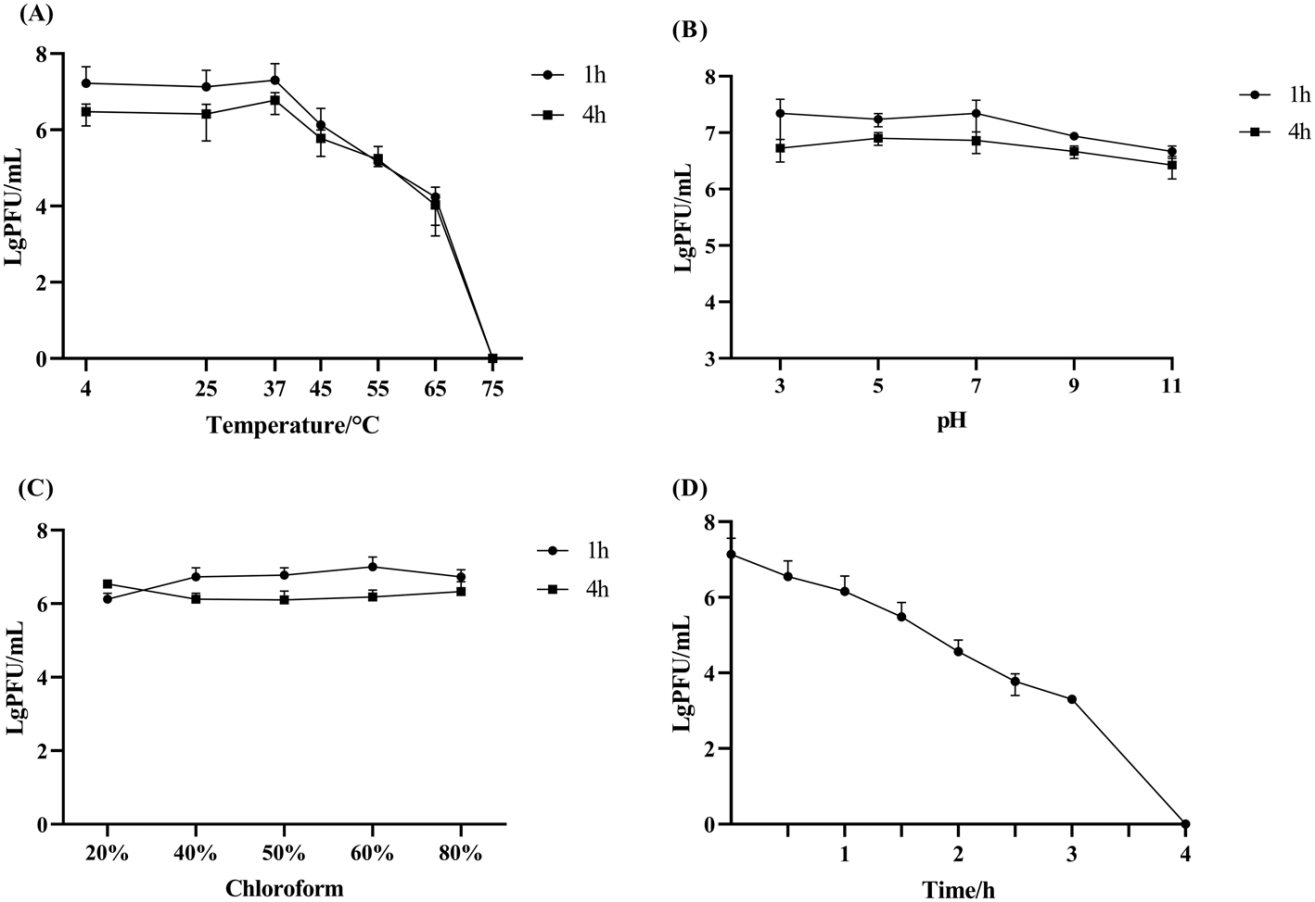
Stability of phage Henu10 under different stresses. **(A)** Thermal stability against various temperatures after 1 and 4 h intervals. **(B)** Impact of wide pH values after 1 and 4 h intervals. **(C)** Sensitivity to different chloroform concentrations after 1 and 4 h intervals. **(D)** Effect of UV radiation.

Phage Henu10’s stability against different temperatures, pH values, chloroform ratios, and ultraviolet irradiation was assessed. Phage Henu10 showed excellent stability over the temperature range of 4 °C to 37 °C, with no significant alterations in titer after 1 h and 4 h of treatment. However, a decline in phage titer was observed from 37 °C to 65 °C. Beyond 65 °C, there was a rapid decrease in phage titer until complete inactivation at 75 °C (**Figure 2A**). In addition, within the pH range of 3 to 11, the phage titer remained relatively stable without significant changes whether treated for 1 h or 4 h (**Figure 2B**). Moreover, when phage Henu10 was treated with different concentrations of chloroform for different times, its titer remained relatively stable (**Figure 2C**). Furthermore, upon prolonged exposure to 40 W UV irradiation, there was a gradual decrease in phage Henu10 titer within 0 to 3 h, followed by a rapid decline to zero after 4 h (**Figure 2D**). These findings highlight the potential for phage Henu10 therapeutic application.

### 2.4 Genome sequencing and bioinformatic analysis

**Figure 3:**
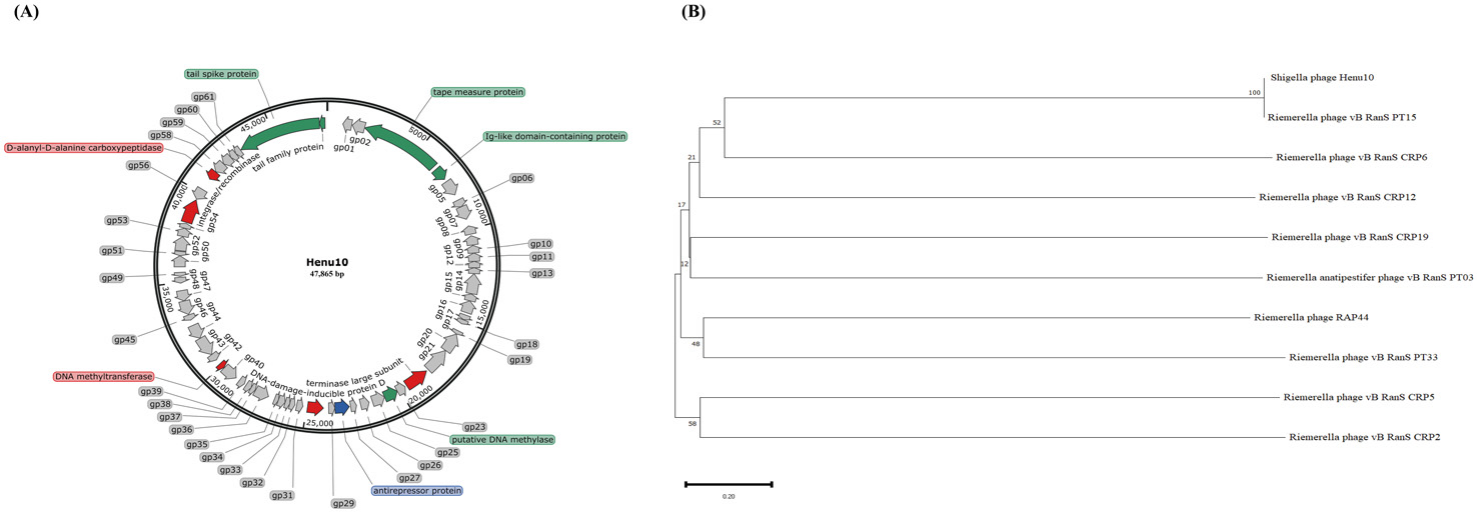
Genome characterization and phylogenetic analysis of Phage Henu10. **(A)** The circular genome map of phage Henu10. **(B)** Phylogenetic tree analysis of phage Henu10.

The genome of phage Henu10 consists of linear double-stranded DNA. Its Genbank accession number is OQ791282.1. The genome sequence comprises 47865 bp, with a GC content of 35.06%. The proportions of the four bases are as follows: A accounts for 28.05%, C for 18.88%, G for 16.18%, and T for 36.9%. The tRNAscan-SE online software did not detect any tRNA (tRNALys). As shown in **Figure 3A**, a total of 65 ORFs were identified, consisting of 13 forward coding ORFs and 52 reverse coding ORFs. Subsequent BLASTP analysis revealed that out of the total 65 ORFs, 52 encoded hypothetical proteins, while the remaining 13 ORFs failed to align with appropriate coding proteins during BLASTP alignment. Among this, one anti-repressor protein was detected. Furthermore, bioinformatics analysis demonstrated that the Henu10 genome exhibits a gene function mosaic structure commonly observed in bacteriophage genomes. Interestingly, no genes coding for antibiotic resistant genes, integrases or bacterial virulence factors were identified in phage Henu10 genome encouraging its use in clinical application (**Figure 3A**).

The BLASTn analysis revealed that only 12 phage species exhibit similarity to Henu10, primarily comprising *Riemerella* phage and *Riemerella anatipestifer* phages. We have identified the top 20 species resembling phage Henu10, encompassing both phages and plasmids. The high bootstrap values near the root of the evolutionary tree also indicate that the phage has high homology with *Riemerella anatipestifer* phage (**Figure 3B**).

### 2.5 Combining sub-lethal antibiotics with phage Henu10 is better than phage and antibiotic alone

Nine antibiotics representing different classes were selected for evaluating PAS, including AMP, CTX, KAN, TCY, CIP, RIF, POL, SDI, and TMP. The MIC values of the studied antibiotics used in this study are shown in **Table 1**. The potential synergistic impact of phage Henu10 in conjunction with sub-MIC values of the tested antibiotics was investigated using a time-killing assay.

**Table 1:** The MICs of antibiotics with different mechanisms of action.

Antibiotic class	Antibiotic	MIC (μg/mL)
Beta-lactams	Ampicillin (AMP)	10
	Cefotaxime sodium (CTX)	0.4
Aminoglycosides	Kanamycin (KAN)	30
Tetracyclines	Tetracycline (TCY)	>50
Sulfonamides	Sulfadiazine (SDI)	>50
Quinolones	Ciprofloxacin (CIP)	0.1
Polymyxins	Polymyxin B (POL)	7.5
Rifamycins	Rifampin (RIF)	2.5
Diaminopyrimidines	Trimethoprim (TMP)	3.75

**Figure 4:**
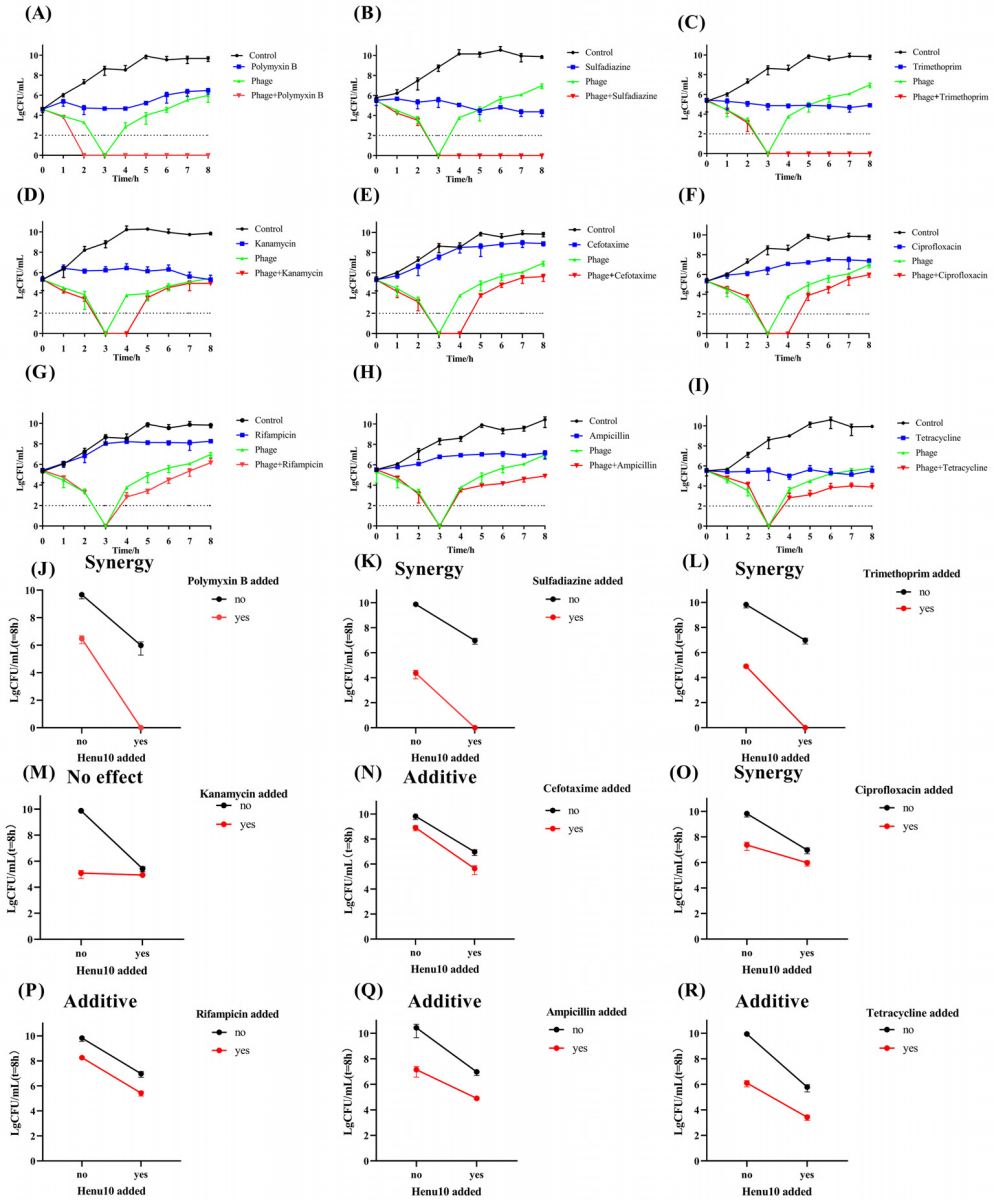
Time-killing assay showing bacteriolytic activity of phage Henu10 in combination with antibiotics. **(A–I)** Polymyxin B, Sulfadiazine, Trimethoprim, Kanamycin, Cefotaxime, Ciprofloxacin, Rifampin, Ampicillin, Tetracycline. Interaction plots of phage Henu10 with antibiotics. **(J–R)** Polymyxin B, Sulfadiazine, Trimethoprim, Kanamycin, Cefotaxime, Ciprofloxacin, Rifampin, Ampicillin, Tetracycline. CFU, colony forming unit. A two-way ANOVA was employed on interaction plots to test for statistical significance.

Combining phage Henu10 with POL (3.75 μg/mL), SDI (50 μg/mL), and TMP (1.875 μg/mL) resulted in more than a 95% reduction in bacterial density. Phage-POL combination treatment was 2.03 times more effective than POL alone and 2.39 times more effective than phage alone. Phage-SDI efficacy was 0.79 times higher than SDI alone and 2.46 times higher than phage alone. Phage-TMP efficacy combination efficacy was twice as much as TMP alone and 2.45 times as much as phage alone. These combinations exhibited a notable ability to inhibit the emergence of resistant bacterial strains (**Figures 4A–C**), suggesting a pronounced synergy that outperformed individual treatments involving either phage Henu10 or antibiotics, as shown by the interaction plots (**Figures 4J–L**).

Moreover, the addition of KAN (15 μg/mL), CTX (0.2 μg/mL), or CIP (0.05 μg/mL) to phage Henu10 resulted in undetectable bacteria for 3 to 4 h. The efficiency of phage-KAN was 0.12 times and 0.11 times more than individual treatment with KAN and phage, respectively. The combination efficiency of phage-CTX was 3.54 times and 0.47 times greater than mono-therapy involving CTX and phage alone, respectively. Phage-CIP was 0.57 times and 0.35 more efficient than CIP alone and phage alone, respectively. These results suggest that KAN, CTX, and CIP in combination with phage Henu10 delayed the emergence of resistant bacteria (**Figures 4D–F**), but the phage-KAN combination yielded no effect, the phage-CTX had additive effect, and the phage-CIP exhibited synergistic effect (**Figures 4M–O**).

Furthermore, the efficacy of phage Henu10 in the presence of RIF and AMP was 1.37 times and 1.10 times higher than each antibiotic alone, respectively, and 0.28 times and 0.96 times higher than phage alone, respectively (**Figures 4G, H**). Both phage-RIF and AMP showed additive interaction (**Figure 4P, Q**). Regarding TCY, its high MIC value makes its use in practical applications challenging. Therefore, a 1/10 MIC (50 ug/mL) of TCY was employed to evaluate PAS. Phage-TCY efficiency was 0.42 times and 0.48 times higher than that of TCY alone and phage alone, respectively (**Figure 4I**). Phage-TCY did not delay the emergence of resistant bacteria, but TCY had an additive effect with phage Henu10 (**Figure 4R**).

### 2.6 Effect of sub-lethal antibiotics on latent period and burst size of phage Henu10

**Figure 5:**
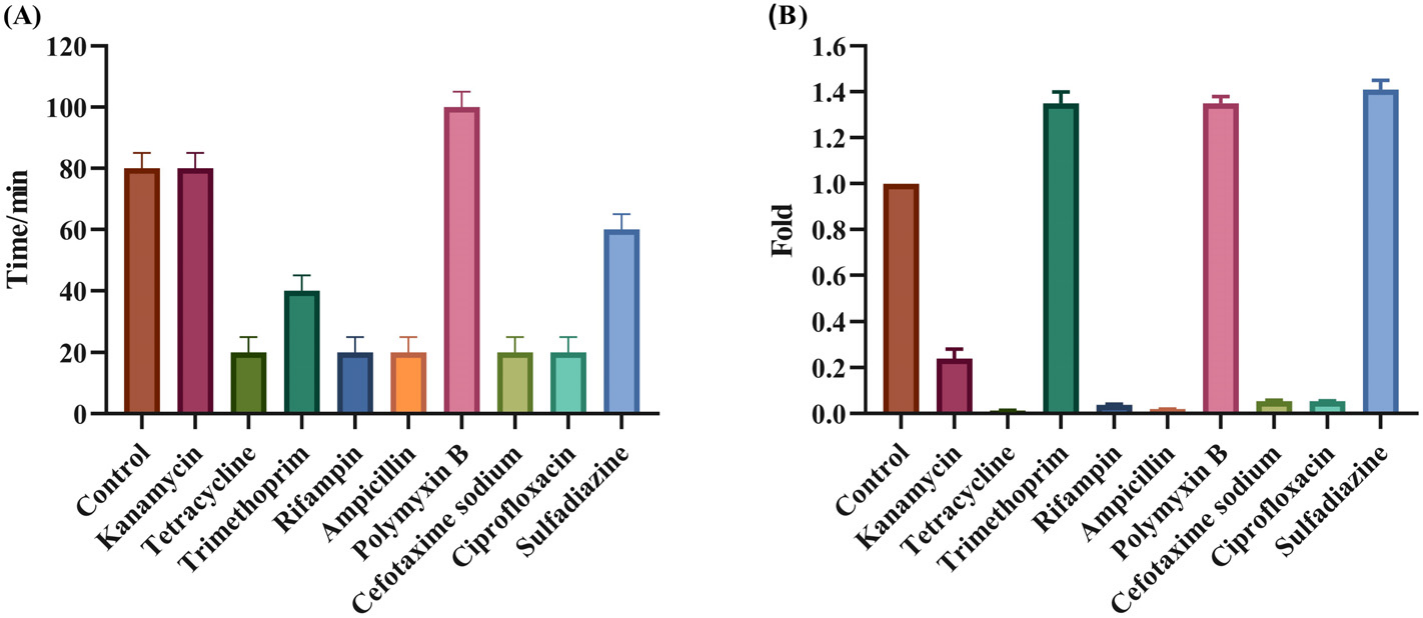
Evaluation of the synergistic effect of phage and antibiotics. Effect of PAS on phage Henu10 latent period **(A)**, and burst size **(B)**.

The effect of PAS on both burst size and latent period of Henu10 phage was also evaluated alongside the ability of PAS. With the addition of POL, the phage latency was delayed by approximately 20 min, while KAN was unchanged. For SDI and TMP, it was shortened by nearly 20 min and 40 min, respectively. However, TCY, RIF, AMP, CTX, and CIP were all shortened by 60 min (**Figure 5A**). Furthermore, with regards to the phage burst size, SDI burst size increased to 1.35-fold, POL and TMP increased to 1.25-fold, and KAN, TCY, RIF, AMP, CTX, and CIP were all decreased by more than 80% (**Figure 5B**).

### 2.7 Screening, identification of phage-resistant strain, and adsorption assay

**Figure 6:**
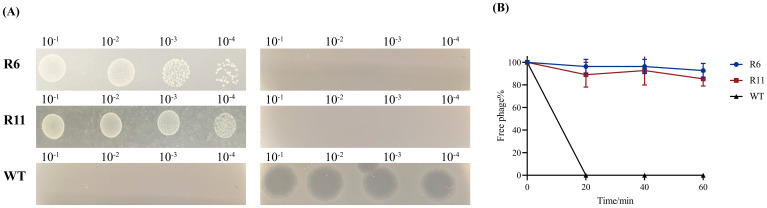
Identification of the phage-resistant strains. **(A)** The identification of phage-resistant strains and the wild type (WT) strain (The standard soft-agar spot assays in the left and inverted spot assays in the right). **(B)** Adsorption rates of wild-type and phage-resistant strains.

The emergence of phage-resistant phenotypes was observed when co-culturing phage Henu10 with its host. No lysis zones were observed in the standard soft-agar spot assay with the phage-resistant strains (R6 and R11), unlike the lysis zones that appeared with the WT strain. In addition, the phage-resistant phenotypes (R6 and R11) exhibited regular growth on inverted spot agar plate, whereas phage-susceptible bacteria showed limited growth (**Figure 6A**). The adsorption of phage Henu10 was assessed by measuring the free phages in the supernatant at specific time intervals. Phage particles was effectively adsorbed onto the cells of the WT strain within 20 min. However, the free phage content for resistant strains R6 and R11 remained at 100%, indicating no phage adsorption (**Figure 6B**).

### 2.8 Phage-resistant strains have increased sensitivity to ciprofloxacin and kanamycin

**Figure 7:**
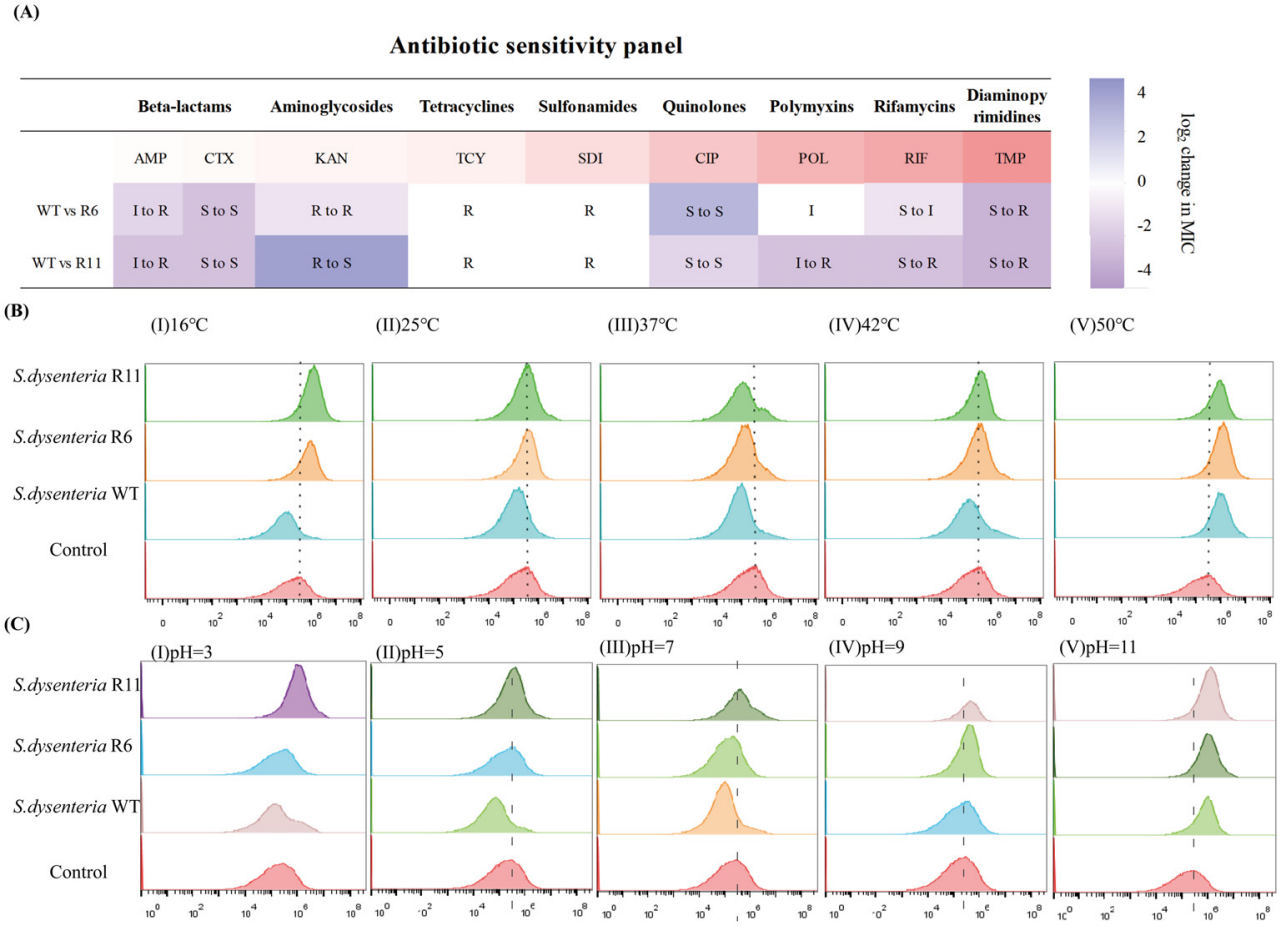
The fitness-cost of phage-resistant strains. **(A)** Antibiotic susceptibility panel of nine antibiotics against the wild type (WT) and phage-resistant strains (R6 and R11). S, sensitive; I, intermediate; R, resistant. Antibiotics: AMP, Ampicillin; CTX, Cefotaxime; KAN, Kanamycin; TCY, Tetracycline; SDI, Sulfadiazine; CIP, Ciprofloxacin; POL, Polymyxin B; RIF, Rifampin; TMP, Trimethoprim. **(B)** Fluorescence imaging of temperature stress response to the wild type (WT) and phage-resistant strains. (I) 16 °C, (II) 25 °C, (III) 37 °C, (IV) 42 °C, and (V) 50 °C. **(C)** Fluorescence imaging of pH stress response to the wild type (WT) and phage-resistant strains. (I) pH=3, (II) pH=5, (III) pH=7, (IV) pH=9, and (V) pH=11.

The susceptibility profile of the phage-resistant strains was determined against nine antibiotics. The R6 and R11 strains exhibited various antibiotic susceptibility profiles as follows: 4x decrease (CIP and KAN), respectively, 5x increase (AMP), 8x and 4x increase (CTX), respectively, 8x increase (TCY, SDI, and TMP), 10x and 5x (RIF), respectively, in MIC relative to the WT strain. Interestingly, the R11 strain became KAN-sensitive, unlike the KAN-resistant WT strain. The clinical interpretation of the MICs of the WT, R6, and R11 strains against the tested antibiotics is shown in **Figure 7A**.

### 2.9 Phage-resistant strains are sensitive to temperature and pH stress

PI is a fluorescent dye that is impermeable to living cells with intact membranes but can permeate dead cells with damaged membranes. PI binds to DNA and emits fluorescence upon excitation, indicating membrane damage and cell death. The flow cytometry analysis showed that the fluorescence intensity of the WT strain demonstrated decreased levels after a 2-h treatment at a temperature range from 16 °C to 50 °C in comparison to the control condition. However, the fluorescence intensity of the R6 and R11 strains increased at 16 °C and 50 °C and decreased at 37 °C. Conversely, there was no significant alteration observed after subjecting the R6 and R11 strains to 25 °C and 42 °C (**Figure 7B**).

Regarding the pH stress, the fluorescence intensity of the WT strain exhibited a decrease at pH levels of 3, 5, and 7, with no substantial alteration observed at pH 9, and an increase noted at pH 11. Conversely, the fluorescence intensity of the resistant strain R6 remained constant at pH values of 3 and 5, decreased at pH 7, and increased at pH 9 and 11. The fluorescence intensity of the R11 strain remained consistent at pH levels of 5 and 7, while showing an increase at pH levels of 3, 9, and 11. This suggests that the WT strain thrived in environments ranging from strong acid to weak alkaline (pH 3-9). In contrast, the R6 strain experienced normal growth and better adapted in strong acid-to-neutral pH environments (pH 3-7), whereas the R11 strain can only grow normally and survive in weak acid-to-neutral pH environments (pH 5-7) as shown in **Figure 7C**. Consequently, these findings indicate that the WT strain demonstrated robust adaptability to varying temperatures or acid-base environments, whereas the resistant strains exhibited sensitivity to such changes.

### 2.10 Reduced biofilm formation and diminished *in vivo* colonization of phage-resistant strains

**Figure 8:**
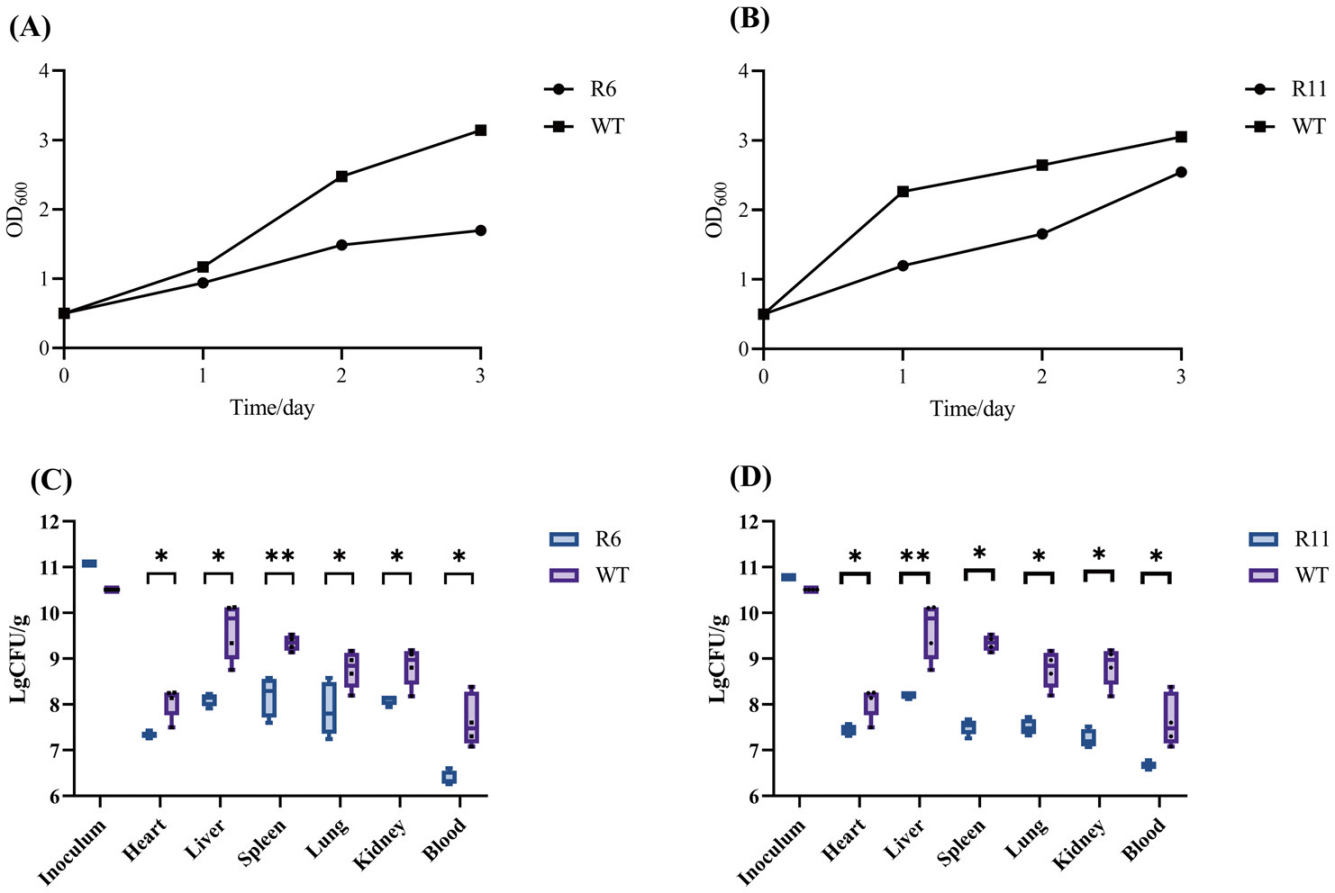
Evaluation of the biofilm formation ability and colonization capability of phage-resistant strains R6 and R11 in mice. **(A)** The biofilm of the resistant strain R6 and the wild-type strain (WT) under the ciprofloxacin stress. **(B)** The biofilm of the resistant strain R11 and the wild-type strain under the kanamycin stress. OD, optical density. *In vivo* fitness of phage-resistant R6 **(C)** and R11 **(D)** S. dysenteriae strains as compared to the wild-type (WT). Following a 12-h period post-infection, the bacterial burden present at various tissue locations in both WT and phage-resistant strains of was adjusted based on the weight of the respective tissues. Each point represents data from one mouse (n= 4; single- tailed Mann–Whitney test, *P<0.05, **P<0.01). CFU, colony forming unit.

The effect of CIP and KAN on the biofilm formation capability of WT and R6 as well as WT and R11 strains, respectively, was evaluated using the crystal violet assay. After treatment with either CIP or KAN, the biofilm-forming capacity of either the R6 or R11 strains was lower than that of the WT strain, respectively. This indicates that the resistant R6 and R11 strains exhibited higher susceptibility to CIP and KAN, respectively, than the WT strain (**Figures 8A, B**).

The *in vivo* fitness of the phage-resistant strains was investigated using a mouse model. Mice groups were infected with R6, R11, or the WT strain. 12 h post-infection, the extent of bacterial colonization was evaluated in mice blood and organs. In the R6 strain, a 2-log reduction in the bacterial load in the heart, spleen, lungs, and kidneys was observed compared to the WT strain. Additionally, the liver and blood bacterial load of the phage-resistant strain were reduced by more than 2 logs. In the R11 strain, there was a 1-log decrease in the bacterial burden in the heart, while the bacterial burden in the other organs decreased by more than 2 logs (**Figures 8C, D**). Consequently, the phage-resistant strains demonstrated lowered fitness *in vivo* as compared to the WT strain.

## 3 Discussion

In this study, a novel lytic *S. dysentery* phage, named Henu10, was successfully isolated and characterized. The bacteriophage Henu10 exhibited promising synergistic interactions when administered alongside a combination of antibiotics including polymyxin B, sulfadiazine, and trimethoprim. Phenotypic characterization of the phage-resistant strains revealed significant fitness cost as a trade-off, which led to increased antibiotic sensitivity to certain antibiotics and environmental stressors, attenuated virulence, and defective colonization in a mouse model.

An intriguing observation is that the phage-resistant strains R6 and R11 exhibit sensitivity to different classes of antibiotics, indicating that these strains possess distinct genetic structures. Therefore, further whole genome sequencing of the phage-resistant strains is necessary to identify the mutated genes using molecular biological methods, which will help elucidate the molecular mechanisms underlying the adaptive trade-offs. Meanwhile, our study found that phage-resistant strains are not always exhibit sensitivity to antibiotics or environmental stress. In fact, we discovered that identifying phage-resistant strains that develop tolerance to antibiotics is easier than identifying those that exhibit sensitivity to antibiotics. As noted in this research, the phage-resistant strains R6 and R11 show enhanced tolerance to trimethoprim and ampicillin (**Figure 7A**) and reduced sensitivity to osmotic pressure compared to the wild-type strains (data not shown). These findings suggest that the clinical implementation of phage-antibiotic combination therapy should rest on a foundation of comprehensive *in vitro* and *in vivo* research.

Some study (Alseth et al., 2019) suggests that *in vitro*, phage-resistant bacterial strains mainly emerge through mutations in their phage adsorption receptors. In contrast, *in vivo*, the development of phage-resistant strains is more significantly influenced by the CRISPR-Cas system. Our research revealed that specific antibiotics, such as sulfadiazine and trimethoprim, have the potential to delay or inhibit the emergence of phage-resistant bacterial strains, although further validation through *in vivo* animal studies is necessary.

In conclusion, the current findings highlight the potential of phage-antibiotic combinations for treating shigellosis while offering valuable insights into fitness trade-offs that could improve the effectiveness of phage therapies against resistant shigellosis in clinical settings.

## 4 Materials and methods

### 4.1 Phage isolation, purification, and propagation

Sewage samples were collected from chicken farms in Kaifeng City, and *S. dysenteriae* was utilized as the host bacteria for the isolation, purification, and propagation of phage Henu10. In brief, 50 mL of sewage samples was centrifuged at 10,000 rpm for 5 min to remove sediment. The supernatant was then filtered using filter paper, followed by further filtration through a sterile 0.22 μm filter. Next, 2 mL of logarithmic phase culture of *S. dysenteriae* was added to a conical flask containing 30 mL of the previously filtrate, followed by incubation in a shaker incubator at 37 °C for 6 h. After centrifuging the mixture at 10,000 rpm for another 5 min, the supernatant was filtered again using a sterile 0.22 μm filter, and a spot assay was employed to detect the presence of phages (Zhang et al., 2021). Phage purification was carried out by picking single-plaque, resuspended in 1 mL LB broth, serially diluted, and plated using the double layer agar (DLA) technique, and this process was repeated three more times (Chen et al., 2018). The purified phage was propagated as previously described (Carey-Smith et al., 2006). The plates with confluent lyses were soaked overnight in saline magnesium (SM) buffer, then decanted into a centrifuge tube along with the scraped upper soft-agar layer. After centrifugation, the supernatant was through a sterile 0.22 μm filter before storage at 4°C.

### 4.2 Transmission electron microscopy

To visualize phage Henu10 particles, aliquots of 20 μL of the purified concentrated phage were dropped onto copper grids, and after 15 min of precipitation, one drop of 2% phosphotungstic acid (PTA) was added to the copper mesh to negatively stain for 10 min. Then, the morphology of the phage was observed under a FEI Tecnai G2 spirit 120 kV TEM (Zhang et al., 2021).

### 4.3 Determination of multiplicity of infection and one-step growth curve

To determine the optimal MOI of phage Henu10, logarithmic phase culture of *S. dysenteriae* was adjusted to a concentration of 2×10^8^ CFU/mL. Henu10 with varying titers, ranging from 2×10^9^ PFU/mL to 2×10^4^ PFU/mL, were mixed with the prepared host bacteria, resulting in MOI values of 10, 1, 0.1, 0.01, 0.001, and 0.0001, respectively. The mixture was incubated at 37 °C for 8 h with shaking. Then, the lysate was centrifuged at 10,000 rpm for 2 min at 4°C, and the supernatant was filtered using a sterile 0.22 μm filter. The phage titer was determined using DLA technique, and the proportion corresponding to the highest plaque forming unit (PFU) value represented the optimal MOI (Zhang et al., 2017). A one-step growth curve of phage Henu10 was conducted as previously described (Ryan et al., 2012). In brief, S. dysenteriae culture in the logarithmic growth phase (2×10^8^ CFU/mL) was infected with phage Henu10 (1×10^5^ PFU/mL) according to the optimal infection ratio. The mixture was incubated at 37 °C with shaking. Every 20 min, a sample of the mixture was taken out and centrifuged at 10,000 rpm for 1 min. The supernatant was subjected to titer determination using the DLA technique.

### 4.4 Thermal, pH, chloroform, and UV radiation stability studies

To assess phage stability against different environmental conditions, phage Henu10 lysate was incubated at different temperatures (4 °C, 25 °C, 37 °C, 45 °C, 55 °C, 65 °C, and 75 °C), pH ranges (pH 3, 5, 7, 9, and 11), and chloroform concentrations (20%, 40%, 50%, 60%, and 80%) for 4 h. Subsequently, samples were collected after 1 and 4 h intervals, underwent a tenfold serial dilution, and the phage titer was calculated using the DLA technique (Wdowiak et al., 2022). To determine the stability of phage Henu10 under UV radiation, a 1 mL aliquot of phage Henu10 lysate was added to a 1.5 mL EP tube and positioned at a distance of 10 cm from a 40 W UV lamp emitting radiation at a wavelength of 253.7 nm. Samples were withdrawn at time intervals of 0.5 h, 1 h, 1.5 h, 2 h, and 4 h to assess the phage titer (Guo et al., 2023).

### 4.5 Phage DNA extraction, genome sequencing, and data analysis

The genomic DNA of the phage was extracted using the protease K- Sodium dodecyl sulfate (SDS) and phenolic extraction method (Xing et al., 2017). Firstly, DNase I was added to the purified phage particles at a final concentration of 5 μg/mL, followed by the addition of RNase A at a final concentration of 1 μg/mL. The mixture was then incubated at 37 °C for 1 h to remove exogenous DNA and RNA. Next, ethylenediaminetetraacetic acid (EDTA; pH 8.0) was added to achieve a final concentration of 20 mmol/L in order to inactivate the previously added DNase I and RNase A. Subsequently, Protease K was added at a final concentration of 50 μg/mL along with SDS at a final concentration of 0.5%. The mixture was inverted and placed in a water bath at 65 °C for 6 h to release the phage DNA from the protein capsid. For extraction, an equal volume of phenolic extraction solution was used under fume hood conditions at -4 °C and centrifuged at 10,000 rpm for 10 min. The upper aqueous phase was retained while discarding other phases. Then, two times the volume of anhydrous ethanol and one-tenth volume of 3 mol/L of sodium acetate (NaAc; pH 5.2) were added before placing it overnight in a refrigerator set to -20 °C. After centrifugation at 4 °C and 12,000 rpm for 20 min, the supernatant was discarded and the precipitate was washed with 70% ethanol followed by anhydrous ethanol. Finally, the extracted DNA was dried and kept at -20 °C until use.

The extracted genomic DNA of phage Henu10 was sent to Shanghai Sangong Biological Co., Ltd. For DNA sequencing, the Illumina NovaSeq6000 sequencing platform was performed. Softberry (http://linux1.softberry.com/berry.phtmltopic=virus0&group=programs&subgroup=gfindv) and GeneMarkTM (http://exon.gatech.edu/GeneMark/) were used to identify the open reading frames (ORFs). MEGA7 software was employed to analyze the neighbor-joining evolutionary tree. Homologous DNA sequence alignment was performed using BLASTn (Altschul et al., 1990; El-Telbany et al., 2021)(http://blast.ncbi.nlm.nih.gov/Blast.cgi) and FASTA (http://www.ebi.ac.uk/Tools/fasta33/index.html). The existence of tRNA in the phage genome was detected using tRNAscan-SE (http://lowelab.ucsc.edu/tRNAscan-SE/). Proksee (https://proksee.ca) was used to map the Henu10 phage genome.

### 4.6 Phage-antibiotic combination

Initially, the minimum inhibitory concentration (MIC) for nine antibiotics from different classes was determined against S. dysenteriae isolate using the broth microdilution method (Li et al., 2021b). The antibiotics included Polymyxin B (POL), Sulfadiazine (SDI), Trimethoprim (TMP), Kanamycin (KAN), Cefotaxime (CTX), Ciprofloxacin (CIP), Rifampin (RIF), Ampicillin (AMP), and Tetracycline (TCY). Subsequently, the possible synergy between phage Henu10 and the tested antibiotics was investigated at their sub-MIC values using a time-killing assay (Irie et al., 1997). In brief, the initial bacterial concentration was 1×10^5^ CFU/mL, and the optimal phage infection rate was 0.001, achieved with an initial phage concentration of 1×10^2^ PFU/mL. The experiment included four groups: control, antibiotic alone, phage alone, and phage and antibiotic combination. Samples were taken from each group every 1 h for a total of 8 h for bacterial colony-forming unit (CFU/mL) counting. A two-way ANOVA was employed on interaction plots to evaluate possible phage-antibiotic synergy (PAS). The impact of PAS on the lytic cycle of Henu10 phage, including burst size and latent period, was also investigated (Ryan et al., 2012). Aliquots were taken from either bacteria/phage or bacteria/phage/antibiotic mixtures for phage titer determination using the DLA technique.

### 4.7 Screening and isolation of phage-resistant strains

Phage-resistant strains were identified through a spot assay (Gordillo Altamirano et al., 2021). Following incubation, the bacterial colonies that appeared in the center of lysis zones were selected and streaked onto agar plates for two rounds for isolation of individual colonies. Confirmation of the phage-resistant phenotypes was performed through standard soft-agar spot assays and inverted spot assays. For standard soft-agar spot assays, phage-resistant (2×10^9^ CFU/mL) and wild-type (WT) *S. dysenteriae* (2×10^9^ CFU/mL) were spread on the agar plate, then the phage Henu10 (1×10^9^ PFU/mL) was serially diluted, and 5 µL aliquots of each dilution were spotted onto the DLA plates pre-seeded with phage-resistant phenotypes and WT bacteria, respectively. After drying, the plates were incubated at 37 °C for 24 h. Phage-resistant phenotypes were confirmed by the absence of lysis zones on the soft-agar overlay. For inverted spot assays, phage Henu10 (1×10^9^ PFU/mL) was evenly spread on agar plate. Then, phage-resistant (2×10^9^ CFU/mL) and WT (2×10^9^ CFU/mL) strains were then diluted and pipetted onto agar plates containing phage Henu10. The plates were incubated at 37 °C for 24 h before being examined for the phage-resistant colonies.

### 4.8 The adsorption assay and the antibiotic susceptibility testing

To determine if the resistant strains could adsorb onto phage Henu10, an adsorption assay was performed for the WT and the phage-resistant strains (R6 and R11). In brief, bacteria and phage Henu10 were co-cultured overnight in LB broth at an MOI of 0.001. The mixture was incubated at 37 °C with shaking. At designated time points, samples were withdrawn, centrifuged at 8,000 rpm for 2 min and the supernatant was diluted to quantify free phage particles (Gordillo Altamirano et al., 2021). The MICs of the nine tested antibiotics were reassessed against the R6 and R11 strains, as mentioned earlier (Li et al., 2021b).

### 4.9 Flow cytometry

The viability of the WT, R6, and R11 strains was assessed in response to various stressors using fluorescence-based flow cytometry (Hong et al., 2020). Briefly, the R6, R11, and WT strains were exposed to different temperatures and pH ranges for 2 h. Then, the cells were collected, washed twice with phosphate-buffered saline (PBS), exposed to 50 μL of propidium iodide (PI) dye, and incubated for 20 min at 37 °C in the absence of light. Following incubation, the fluorescence intensity was quantified using a CytoFLEX flow cytometer (Becman Coulter Bioscience, USA). A total of 100,000 cells were analyzed at a rate of 35lL/min for each sample to determine fluorescence. Detection parameters were PE channel. Data were analyzed using FlowJo software.

### 4.10 Biofilm experiment

A crystal violet assay was utilized to monitor the biofilm-forming capacity of the R6 strain and WT strain when subjected to ciprofloxacin (0.005 μg/mL), as well as the R11 strain and WT strain when subjected to kanamycin (3.75 μg/mL) was assessed at 24-h intervals over a total of 72 h, and the optical density (OD) was measured at 600 nm (Gordillo Altamirano et al., 2021).

### 4.11 *In vivo* mouse model

To assess the colonization potential of the WT and the phage-resistant strains, a mouse infection model was conducted (Gordillo Altamirano et al., 2021). Four females, 6- to 10-week-old BALB/c mice, were used per group. Bacterial inoculums, either wild-type or phage-resistant *S. dysenteriae*, were prepared at a concentration of 10 (Deris et al., 2013) CFU in 100 μL 1×PBS and injected intraperitoneally into mice. The mice were monitored for up to 12 h, blood samples were obtained through heart puncture, and subsequently, mice were sacrificed and the heart, liver, lung, right kidney, and spleen were collected. The organs were then weighed and homogenized using PBS. Subsequently, the bacterial density in blood and tissue samples was quantified through a process of serial dilution. The animal experiments involved in this study have been approved by the Biomedical Research Ethics Subcommittee of Henan University, with the approval number: HUSOM2024-616.

### 4.12 Statistical analysis

Statistical analysis was performed using GraphPad Prism 9.5.1 software using t-tests unless otherwise stated. At least three biological replicates were conducted for each experiment. Each data point represents the average of independent replicate experiments, and the plotted values indicate the mean ± standard deviation.

## Data Availability

The data presented in the study are deposited in the NCBI repository, accession number OQ791282.1.
